# Establishment of a medium-scale mosquito facility: tests on mass production cages for *Aedes albopictus* (Diptera: Culicidae)

**DOI:** 10.1186/s13071-018-2750-7

**Published:** 2018-03-19

**Authors:** Dongjing Zhang, Yongjun Li, Qiang Sun, Xiaoying Zheng, Jeremie R. L. Gilles, Hanano Yamada, Zhongdao Wu, Zhiyong Xi, Yu Wu

**Affiliations:** 1 Department of Parasitology, Zhongshan School of Medicine, Sun Yat-sen University, Guangzhou, Guangdong 510080 China; 2 Key Laboratory for Tropical Disease Control, Ministry of Education, Sun Yat-sen University, Guangzhou, Guangdong 510080 China; 3 Guangdong Provincial Engineering Technology Research Center for Diseases-vectors Control, Sun Yat-sen University, Guangzhou, Guangdong 510080 China; 4 Zhongshan School of Medicine, Sun Yat-sen University - Michigan State University Joint Center of Vector Control for Tropical Diseases, Guangzhou, Guangdong 510080 China; 5 Insect Pest Control Laboratory, Joint FAO/IAEA Division of Nuclear Techniques in Food and Agriculture, A1130 Vienna, Austria; 6 Department of Microbiology and Molecular Genetics, Michigan State University, East Lansing, MI 48824 USA

**Keywords:** Mosquito factory, Adult mass-reared methods, Mass production cage, *Aedes albopictus*

## Abstract

**Background:**

Mass egg production is an important component of *Aedes albopictus* mosquito control programs, such as the sterile insect technique and incompatible insect technique, which requires the releases of large number of sterile males. Developing standard operating procedures and optimized cages for adult maintenance of *Ae. albopictus* can improve the mass rearing efficiency.

**Methods:**

Three different sex ratios of females to males with a total number of 4,000 mosquitoes were tested by evaluating the insemination rate, egg production (total number of eggs per cage), female fecundity and egg hatch rate in small cage (30 × 30 × 30 cm). Blood meals with adenosine triphosphate (ATP, 0.05 g/ml), cage structures (Big cage A: 90 × 30 × 30 cm; Big cage B: 90 × 30 × 50 cm or 90 × 50 × 30 cm) and rearing densities (12,000, 16,000 and 20,000 mosquitoes, corresponding to 0.9 cm^2^/mosquito, 0.675 cm^2^/mosquito and 0.54 cm^2^/mosquito, respectively) were also tested and evaluated on the basis of egg production, female fecundity and egg hatch rate. An adult rearing unit holding 15 of Big cage A with optimal egg production was designed to produce 10 million eggs per rearing cycle in a 1.8 m^2^ space.

**Results:**

Female to male ratios at 3:1 in small cages resulted in higher egg production but did not affect insemination rate, female fecundity and egg hatch rate. A concentration of 0.05 g/ml of ATP added to blood meals improved the blood-feeding frequency and thus increased the overall egg production per cage. Cage structures affected the egg production per cage, but not egg hatch rate. A medium rearing density at 0.675 cm^2^/mosquito (16,000 mosquitoes) resulted in higher egg production compared to both low and high densities. An adult rearing unit for *Ae. albopictus* on the basis of Big cage A has been developed with the capacity of producing 10 million eggs within 15 days.

**Conclusions:**

Our results have indicated that the adult rearing methods and adult maintenance unit are recommended for *Ae. albopictus* mass rearing in support of the establishment of a medium-sized mosquito factory.

**Electronic supplementary material:**

The online version of this article (10.1186/s13071-018-2750-7) contains supplementary material, which is available to authorized users.

## Background

As a major vector for arbovirus diseases such as dengue, chikungunya and Zika, *Aedes albopictus* is responsible for a huge burden to public health worldwide [1]. The current control strategies relying on the reduction of breeding sites and insecticide application are unsustainable and can become an economic and environmental burden on both public administration and human health [2, 3]. Vector control strategies such as the sterile insect technique (SIT), the incompatible insect technique (IIT), or a combination of both techniques, are currently under development for mosquito control [4]. Both the SIT and IIT are species-specific and environmental friendly approaches for vector control and are based on the induction of sterility into the natural population by releasing a large number of irradiation-sterilized or *Wolbachia*-incompatible males [5]. *Aedes albopictus* has been considered to be a suitable candidate for large suppression programs [6], and the feasibility of such a suppression program has been successfully tested in past field trials using either SIT [7] or IIT [8]. As stated by the WHO in March 2017, the combined SIT/IIT technology has potential for long-term control of *Ae. albopictus* and *Aedes aegypti* mosquitoes [9].

As any large-scale suppression program relies on continuous releases of a large number of competitive sterile males, efficient and effective approaches for mass rearing the target insects is essential. In a mosquito factory, mass rearing can be divided into two independent parts: rearing for male releases and rearing for colony egg production [10]. The latter should not only provide a sufficient number of eggs for male releases but also for colony maintenance. For *Ae. albopictus* mass rearing, 90% of eggs may be used to produce males for releases and 10% for colony maintenance. Many studies have been performed to develop standardized rearing methods for *Ae. albopictus* at the egg [11, 12] or larval stage [13–17]. However, few studies have been performed to develop optimal rearing methods for *Ae. albopictus* at the adult stage, especially under mosquito mass rearing conditions. In addition, selecting a suitable cage structure for rearing adults is also a key for mass egg production. A prototype of a mass production cage (MPC), based on the cage design used for Medflies in Guatemala, which has been modified to allow blood-feeding, sugar-feeding and oviposition for simplified collection of the eggs, has been tested for both *Ae. albopictus* [18] and *Anopheles arabiensis* [19]. The MPC cage was designed to reduce handling and the opening of the cages during operation such as for blood-feeding and egg collection, but the egg production of *Ae. albopictus* per cage was found to be quite low (average 16 eggs per female per blood meal). The cage structure seems to be the main factor resulting in low egg production; for example, the position of the two blood-feeders were positioned to one side of the cage which may cause a reduced blood-feeding rate as the mosquitoes on the other side may not have been attracted by the blood source. In addition to cage structure, mosquito density is another important factor that will influence the egg production. The number of adults per cm^2^ of vertical resting surface in a cage, defined as density-resting surface (DRS), is generally reported to be an important parameter for mating, feeding and longevity and a DRS value of 1.8 (1.8 cm^2^/adult) is considered suitable for mosquitoes maintenance in the laboratory [20]. Mamai et al. [19] found decreased *An. arabiensis* egg production when overloading the cage with pupae (20,000 pupae, DRS ≈ 2.0 cm^2^/adult) compared to cages with optimized density (15,000 pupae). However, for *Aedes* mosquitoes, it is reported that they can be maintained at a relative high density in laboratory conditions. A DRS value of 0.9 is used for *Ae. aegypti* mass production in Brazil [10]. Thus, it is important to develop standardized operation methods and cages for adult maintenance in any mosquito factory.

In this study, three sex ratios of females to males in small cages were evaluated to find an optimal sex ratio for adult maintenance. In addition, blood meals supplemented with ATP were tested for its impacts on egg production, female fecundity and egg hatch rate. Moreover, three different structures of large adult rearing cages were assessed by evaluating the egg production and egg hatch rate, with the aim to find a suitable cage structure and size for adult mass rearing. Lastly, egg production under three different mosquito densities was compared to find an optimal density for adult mass rearing. Based on these results, we discuss the design of an adult rearing unit for *Ae. albopictus* adults in terms of mass egg production in support of establishing a medium-sized mosquito factory.

## Methods

### Mosquito strain and rearing conditions

The triple *Wolbachia*-infected *Ae. albopictus* (HC) strain was used in this study [21]. The HC line has been maintained in the mass rearing factory in Guangzhou, China for three years and experiments were conducted in a climate-controlled room at 27 ± 1 °C, 80 ± 10% RH, and a photoperiod of 12:12 h (L:D). The HC strain was reared as previously described [17].

### Effects of adults sex ratios on insemination rate, egg production, female fecundity and egg hatch rate

**Fig. 1:**
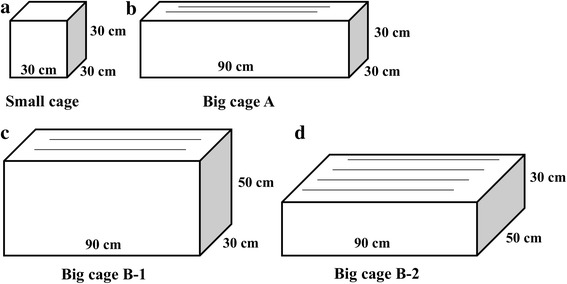
Different dimensions of stainless steel cages for the maintenance of mosquito adults. **a** Small cage (30 × 30 × 30 cm). **b** Big cage A (90 × 30 × 30 cm). **c** Big cage B-1 (90 × 30 × 50 cm). **d** Big cage B-2 (90 × 50 × 30 cm). Black lines represent the number of nets added to increase the overall resting surface for the mosquitoes. The bottom of the cages is stainless steel while the other four sides (except for the right side, light grey part ) are stainless steel mesh sieve (40 sieves/cm^2^). A white blazer with cuffs (diameter 12 × 25 cm) is hung on the right side, allows for placing and removing the oviposition cup

Three different sex ratios of females to males with a total number of 4000 mosquitoes were tested in small stainless steel cages (L × W × H = 30 × 30 × 30 cm, volume = 27,000 cm^3^, Density-Resting Surface (DRS) = 3600 cm^2^, Fig. 1a): 1:1 (2000:2000), 2:1 (2667:1333) and 3:1 (3000:1000) ratios. Pupae were sexed and counted individually and then transferred into each cage for eclosion. Cotton soaked in 10% sugar solution was placed on top of the cages and provided to adults *ad libitum*. Thirty females (three to four days old) were randomly selected from each ratio to check the insemination status under the microscope before blood-feeding [22]. The blood meals were prepared on an aluminum plate covered with Parafilm [10] and were provided to female adults 5–6 days post-eclosion. Oviposition cups were placed into each cage 48 h after blood-feeding. Eggs were collected for 48 h and allowed to mature for 5–6 days. The second blood meal was provided to females after the first egg collection and eggs were collected as described above. The schedule of the experimental rearing procedure for adult *Ae. albopictus* is shown in Additional file 1: Figure S1. Eggs were brushed by soft brush and weighed, and then hatched in hatch solution (1000 ml filtered water containing 0.12 g yeast powder) for 24 h. Egg hatch rate was assessed under the stereoscope as previously described [21, 23, 24]. Five replicates were conducted for each sex ratio.

### Effects of ATP on egg production, female fecundity and egg hatch rate

To assess the effects of blood meals supplemented with adenosine triphosphate (ATP, Sigma-Aldrich, St. Louis, USA) on egg production, individual female fecundity and egg hatch rate, a 0.05 g/ml concentration of ATP was added to the blood meals. The small cages holding 4000 mosquitoes with the 3:1 sex ratio (females:males) were tested for effects of ATP on the reproductive parameters of females. Blood meals without ATP were used as controls. Three replicates were performed for both treatment and control cages.

To determine the reason how ATP may enhance egg production, the female blood-feeding rate, volume, fecundity and egg hatch rate were additionally assessed: fifty fasting female adults (5–6 days old, not engorged with a recent sugar meal, assessed by personal observation) were selected, anaesthetized and measured with an analytical balance. Then females were placed into an empty cage (30 × 30 × 30 cm) for recovery. After 3–4 h, blood meals with ATP were provided to the females. Blood meals without ATP were used as negative control. After 1 h, all of the females in each cage were collected and the number of engorged females was recorded. females Three replicates were performed for both treatment and control cages. Thirty randomly selected engorged were anaesthetized and individually weighed using an analytical balance and the other thirty engorged females were placed into individual oviposition tubes with moist paper for egg collection. The individual female fecundity and fertility were assessed as described above.

### Effects of cage structure on egg production, female fecundity and egg hatch rate

Three different structures of large stainless steel cages were used: Big cage A (Fig. 1b), Big cage B-1 (Fig. 1c), and Big cage B-2 (Fig. 1d). Cage specifications of the three cages tested are shown in Table 1. The Big cage B-1 and Big cage B-2 were the same cage design but set up in different orientation (vertically for B-1 and horizontally for B-2) to test if the inversion of height and width of the cage would affect the egg production.

**Table 1:** Cage specifications of the three tested cages

Cage	L × W × H (cm)	Volume (cm^3^)	Original DRS (cm^2^)	Increased DRS (cm^2^)	Final DRS (cm^2^)
Big cage A	90 × 30 × 30	81,000	7200	3600^a^	10,800
Big cage B-1	90 × 30 × 50	135,000	12,000	6000^b^	18,000
Big cage B-2	90 × 50 × 30	135,000	8400	9600^c^	18,000

^a^Added two pieces of (15 × 60 cm) nets inside the cage to increase the DRS

^b^Added two pieces of (25 × 60 cm) nets inside the cage to increase the DRS

^c^Added four pieces of (20 × 60 cm) nets inside the cage to increase the DRS

*Abbreviations*: *L* length, *W* width, *H* height, *DRS* density-resting surface

The adult maintenance ratio of females to males was 3:1 for all of the following experiments. As described above, each small cage contained 3000 female and 1000 male mosquitoes, corresponding to 6.75 cm^3^/mosquito (calculated by volume) and 0.9 cm^2^/mosquito (calculated by DRS). To attain the same rearing density indices for both Big cages A and B, 9000 female and 3000 male mosquitoes, or 15,000 female and 5000 male mosquitoes were placed in Big cages A and B, respectively (volume = 6.75 cm^3^/mosquito and DRS = 0.9 cm^2^/mosquito). The rearing schedule and data recorded were the same as described above.

### Effects of adult rearing density on egg production, female fecundity and egg hatch rate

**Fig. 2:**
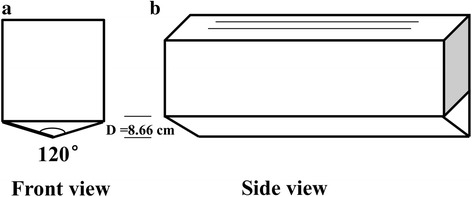
Front view and side view of Big cage A with the stainless steel, V-shaped base. **a** Front view of the stainless steel base with an angle of 120° and a depth of 8.66 cm. **b** Side view of Big cage A with v-shaped base. The two black lines in Big cage A represent the two added pieces of nets to increase the overall resting surface for adults

Big cages A with a 120° “v” angle stainless steel base, which was used for holding the pupae for eclosion and subsequently for draining out any dead pupae after emergence, was used in this study (Fig. 2). Two pieces of (15 × 60 cm) nets were added to the inside of the cage to increase the DRS to 10,800 cm^2^ as described above. Three adult rearing densities at a maintenance ratio of 3:1 (females:males were tested: 12,000 mosquitoes (corresponding to DRS = 0.9 cm^2^/mosquito, low rearing density), 16,000 mosquitoes (DRS = 0.675 cm^2^/mosquito, medium rearing density) and 20,000 mosquitoes (DRS = 0.54 cm^2^/mosquito, high rearing density). Blood meals with ATP were provided to females in each cage. The rearing schedule and data recorded were the same as described above.

### Data collected and statistical analysis

Females were considered to be successfully inseminated if one or more spermathecae was filled with sperm. The insemination rate was recorded as the number of females successfully inseminated divided by the total number of females tested. The egg production per cage was calculated as the sum of the eggs produced from the first and second gonotrophic cycles. The relationship between the number of eggs and their weight for *Ae. albopictus* HC strain was: 150,000 = 1 g eggs, which was close to figures described in our previous study [11]. The female fecundity per cage was calculated as the egg production per cage divided by the number of females placed inside each cage, where we ignored the non-blood-fed and dead females during rearing. Individual female fecundity was calculated from the engorged females. The blood-feeding volume per female was calculated as the weight of engorged females minus the mean weight of fasting females, and then divided by the density of blood with ATP.

Data analysis was conducted using IBM SPSS 20.0 and Graph Pad Prism 6.0 software. To compare the insemination rates, egg hatch rates and blood-feeding rates, the values were first arcsin transformed. ANOVA analysis and Tukey *post-hoc* test were used to compare the egg production, female fecundity and egg hatch rates. Student *t*-test or Mann Whitney U-test were used to compare the egg production, blood-feeding rates, blood volume, individual female fecundity and egg hatch rates between blood meals with and without ATP. The results were presented as (mean ± SEM) in this study.

## Results

### Effects of sex ratio on insemination rate, egg production, female fecundity and egg hatch rate

Under the same rearing density (DRS), the higher the number of female mosquitoes maintained in one cage, the more eggs will be produced, which is beneficial for mass rearing efficiency. Thus it is recommended to stock the cage with a higher ratio of females to males in each cage, but without affecting the insemination rate. In this study, we first tested effects of different sex ratios of females to males on insemination rates (1:1, 2:1 and 3:1) and we observed the three tested ratios resulted in similar insemination rates in the small cage, with more than 96% females that had been successfully inseminated by males (Table 2). As we expected, higher total egg production per cage was observed correlating with the increased number of females introduced into cages (*F*_(2, 12)_ = 38.3, *P* < 0.0001). For example, an average of 37.9% higher egg production was achieved at a 3:1 ratio when compared to a 1:1 ratio (Table 2). However, as the number of female mosquitoes maintained in the cages were different, the individual female fecundity (total egg production divided by the number of females introduced into the cage) showed no significant difference between the three tested ratios (*F*_(2, 12)_ = 2.72, *P* = 0.1063). Similarly, we observed no significant difference on egg hatch rate between the three tested ratios (*F*_(2, 12)_ = 0.11, *P* = 0.8988). These results indicate that a female biased ratio (of up to 3:1) in adult cages will improve egg production without negative impacts on female insemination rate, individual female fecundity and egg hatch rate (Table 2).

**Table 2:** Insemination rate, egg production, female fecundity and egg hatch rate from varied sex ratios of *Aedes albopictus* adults in small cages

Sex ratio (female: male)	Insemination rate (%)	Egg production per cage (10^4^)	Female fecundity	Egg hatch rate (%)
1:1 (2000: 2000)	98.7 ± 0.8^a^	10.3 ± 0.4^a^	51.7 ± 2.0^a^	87.5 ± 0.7^a^
2:1 (2667: 1333)	97.3 ± 1.2^a^	12.8 ± 0.2^b^	48.1 ± 0.7^a^	88.0 ± 1.2^a^
3:1 (3000: 1000)	96.7 ± 1.1^a^	14.2 ± 0.3^c^	47.5 ± 1.1^a^	87.8 ± 0.7^a^

Within each column, values followed by different superscript letters were statistically different in the same treatment cage using ANOVA analysis and Tukey *post-hoc* test (*P* < 0.05)

### Effects of ATP on egg production, female fecundity and egg hatch rate

To improve egg production per cage, we added ATP to stimulate female blood-feeding. As shown in Table 3, an average of 30.0% higher egg production and female fecundity were both observed in small cages where females were provided with 2 blood meals supplemented with ATP (egg production: *t* = 3.37, *df* = 4, *P* = 0.028; female fecundity: *t* = 3.37, *df* = 4, *P* = 0.028), which indicated that an average of 15% higher egg production could be achieved with one blood-feeding with ATP. There was no significant difference between hatch rates in eggs produced by females that were fed on blood meals with and without ATP (*t* = 0.77, *df* = 4, *P* = 0.487).

**Table 3:** Egg production, female fecundity and egg hatch rate of *Aedes albopictus* following blood meals with and without ATP

Treatment	Sex ratio (female: male)	Egg production per cage (10^4^)	Female fecundity	Egg hatch rate (%)
W/O ATP	3000:1000 (3:1)	14.3 ± 1.0^a^	47.5 ± 3.4^a^	88.9 ± 2.9^a^
W ATP	3000:1000 (3:1)	18.6 ± 0.8^b^	61.9 ± 2.6^b^	86.7 ± 1.1^a^

Within each column, values followed by different superscript letters were statistically different according to the Student *t*-test (*P* < 0.05)

*Abbreviations*: *W/O ATP* blood meals without ATP, *W ATP* blood meals with ATP (0.05 g/ml)

**Fig. 3:**
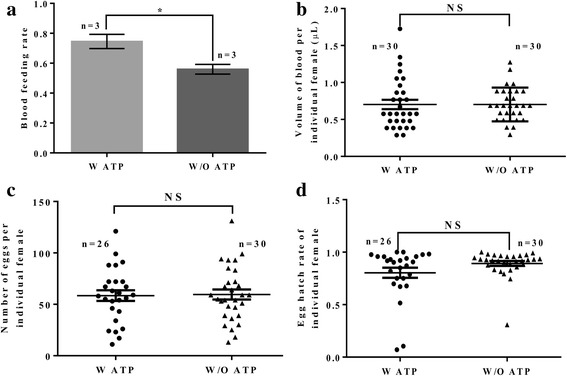
Effects of ATP on blood-feeding rate, blood-feeding volume, female fecundity and egg hatch rate. **a** Blood-feeding rate (W ATP and W/O ATP: *n* = 3). **b** Blood-feeding volume (W ATP and W/O ATP: *n* = 30). **c** Individual female fecundity (W ATP: *n* = 26; W/O ATP: *n* = 30). **d** Individual female egg hatch rate (W ATP: *n* = 26; W/O ATP: *n* = 30). The light gray column or dots represent W ATP; the dark gray column or triangles represent W/O ATP. * indicates statistical difference, Student’s *t*-test, *P* < 0.05; NS indicates no statistical difference, according to the Mann Whitney U-test, *P* > 0.05. *Abbreviations*: W ATP, blood meals with ATP; W/O ATP, blood meals without ATP

To further investigate the causative reason for the ATP-associated increase in egg production, we repeated the above experiment with one blood-feeding and measured blood-feeding rate, blood-feeding volume, individual female fecundity and egg hatch rate. As shown in Fig. 3a, a significant difference was observed on blood-feeding rates of females when providing blood meals with ATP (74.5%) and without ATP (56.0%) (*t* = 3.23, *df* = 4, *P* = 0.030): there was an approximately 18.5% increase in the feeding rate on blood with ATP compared to without ATP. The improved blood-feeding rate correlated with the above-mentioned improvement in egg production. However, no significant differences were observed in blood-feeding volume (*U* = 375.0, *P* = 0.2701, Fig. 3b), individual female fecundity (*U* = 389.5, *P* = 0.9967, Fig. 3c) and egg hatch rate (*U* = 323.0, *P* = 0.2751, Fig. 3d) between blood meals with ATP and without ATP. These results indicate that ATP improves female feeding rate and thus increases egg production per cage.

### Effects of cage structure on egg production, female fecundity and egg hatch rate

Compared to smaller cages for mosquito maintenance, the larger ones can reduce human handling and improve mass rearing efficiency. In this study we set up three different big cage structures (Fig. 1b-d) to test the effects of cage shape on egg production, female fecundity and egg hatch rate, aiming to find a suitable cage structure for mosquito maintenance. Among the three tested cages, Big cage A, Big cage B-1 and Big cage B-2, the latter cage design resulted in the highest egg production (egg production: *F*_(2, 6)_ = 29.3, *P* = 0.0008) while Big cage A resulted in the highest individual female fecundity (*F*_(2, 6)_ = 139.0, *P* < 0.0001) (Table 4). No significant difference was observed on egg hatch rate among these three cage structures (*F*_(2, 6)_ = 1.68, *P* = 0.2633) (Table 4).

**Table 4:** Egg production, female fecundity and egg hatch rate from different *Aedes albopictus* adults rearing cage

Cage structure	No. of mosquitoes	Egg production per cage (10^4^)	Female fecundity	Egg hatch rate (%)
Big cage A	12,000	53.3 ± 1.1^a^	59.2 ± 1.2^a^	90.4 ± 0.2^a^
Big cage B-1	20,000	46.6 ± 1.7^a^	31.1 ± 1.1^b^	88.6 ± 1.1^a^
Big cage B-2	20,000	63.6 ± 1.9^b^	42.4 ± 1.3^c^	87.9 ± 1.3^a^

Within each column, values followed by different superscript letters were statistically different in the same treatment cage according to ANOVA analysis and Tukey *post-hoc* test (*P* < 0.05)

Interestingly, mosquitoes in Big cage B-1 and B-2 were controlled under the same rearing methods and density (DRS/mosquito and volume/mosquito), however, when Big cage B-1 was placed horizontally (referred to as Big cage B-2), an average of 36.5% higher egg production and female fecundity was observed (Table 4). In this study the egg production of Big cage B-2 was 19.3% higher than that of Big cage A, and this improvement in egg production was attributed to 4,500 more female mosquitoes which were placed in the former cage. However, when calculated into individual female fecundity, an average of 39.6% higher female fecundity was obtained in Big cage A compared to Big cage B-2 (Table 4). Taken together, these results indicate that cage structure (height and width) can influence egg production or individual female fecundity, but not egg hatch rate.

### Effects of adult rearing density on egg production, female fecundity and egg hatch rate

Based on our results described above, we selected Big cage A as the potential cage structure for mosquito adult maintenance as it induced the highest level of individual female fecundity (Table 4). With the rearing methods (sex ratio and two blood-feedings with ATP) developed previously, we tested the effects of rearing density on egg production, female fecundity and egg hatch rate by using Big cage A. Three rearing densities were tested with Big cage A: 12,000 mosquitoes, 16,000 mosquitoes and 20,000 mosquitoes, corresponding to 0.9 cm^2^/mosquito (low density), 0.675 cm^2^/mosquito (medium density) and 0.54 cm^2^/mosquito (high density). As shown in Table 5, the medium density resulted in the highest egg production compared to both low and high densities (*F*_(2, 7)_ = 8.18, *P* = 0.0147). An average of 20.8% higher egg production was observed with the medium density compared to low density as there were 4000 more mosquitoes placed in the medium density cage, however, there was a significant difference in female fecundity between medium and low densities (Table 5). Female fecundity decreased under the high rearing density compared to low and medium densities (*F*_(2, 7)_ = 49.3, *P* < 0.0001) (Table 5). These three tested rearing densities had no significant impacts on egg hatch rate (*F*_(2, 7)_ = 3.58, *P* = 0.0852) (Table 5). Our results show that medium rearing density with Big cage A is recommended for adult maintenance as higher egg production is achieved but without affecting female fecundity and egg hatch rate.

**Table 5:** Effects of adult rearing density on egg production, female fecundity and egg hatch rate using Big cage A

No. of mosquitoes	DRS (cm^2^ / adult)	Egg production per cage (10^4^)	Female fecundity	Egg hatch rate (%)
12,000	0.900	60.6 ± 2.1^a^	67.4 ± 2.3^a^	85.1 ± 1.7^a^
16,000	0.675	73.2 ± 2.6^b^	61.0 ± 2.1^a^	85.3 ± 1.5^a^
20,000	0.540	58.8 ± 3.1^a^	39.2 ± 2.1^b^	78.5 ± 2.4^a^

Within each column, values followed by different superscript letters are statistically different in the same treatment cage using ANOVA analysis and Tukey *post-hoc* test (*P* < 0.05)

*Abbreviations*: *DRS* density-resting surface

**Fig. 4:**
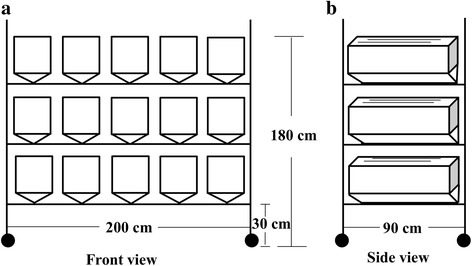
*Aedes albopictus* adults rearing unit. **a** Front view of unit. The unit is 200 cm long and 180 cm in height. The distance between the floor and the first shelf of the unit is 30 cm. This unit can hold 15 Big cages A with stainless steel v-shaped bases. **b** Side view of the unit. The width of unit is 90 cm. The two black lines in Big cage A represent the two added pieces of nets to increase the overall resting surface for adults

With an average production of approximately 0.7 million *Ae. albopictus* eggs at a density of 16,000 mosquitoes for Big cage A, an adult rearing unit was designed for holding 15 Big cages A with the capacity of producing 10 million eggs per rearing cycle, while only occupying a 1.8 m^2^ space (Fig. 4). A comparison between the adult rearing in a Small cage unit (holding 12 small cages) and a Big cage A unit for producing 10 million eggs is shown in Additional file 2: Table S1. The advantage of the Big cage A unit compared to the Small cage unit is the minimized number of cages required (15 *vs* 60), indicating less labor (stocking cages with pupae and subsequent removal of pupal emergence cups from cages, sugar-feeding, blood-feeding, egg collection and cage cleaning, etc.) needed by using the Big cage A unit when mass rearing *Ae. albopictus* in a factory setting.

## Discussion

In this study, we assessed the rearing method and cage structure for *Ae. albopictus* adults in support of the establishment of a medium-scale mosquito facility for the SIT/IIT strategy. Our results indicate that the optimal adult colony maintenance sex ratio lies at 3:1 of females to males. Blood meals supplemented with ATP can improve the egg production as ATP can increase the blood-feeding rate of the females. Cage structure and rearing density can also affect the egg production and overall female fecundity. Big cage A is suitable for adult mass rearing with an achievable egg production of more than 0.7 million eggs per two blood-feedings per rearing cycle. An adult rearing unit for *Ae. albopictus* based on 15 Big cage A has also been developed with the capacity of producing 10 million eggs per rearing cycle as determined by this study.

*Aedes albopictus* male mosquitoes can mate with several females in their lifetime, while it has been suggested that most of the females usually mate only one time [25–27]. Thus, higher egg production per cage can be achieved by increasing the number of females while maintaining the insemination rate. More than 96% insemination rate is observed in the three tested adult maintenance ratios 1:1, 2:1 and 3:1 (Table 2). Considering that a higher egg production is achieved at a 3:1 ratio of females to males compared to the other two ratios (Table 2), this maintenance ratio is recommended for *Ae. albopictus* adult mass rearing. Previous studies have reported that a 3:1 ratio of females to males is also suitable for *Ae. ageypti* mass rearing [10]. For *Ae. albopictus*, males are always seeking to copulate with females, thus a higher ratio of males (e.g. females to males at 1:1) will exacerbate harassment of females, resulting in decreased blood-feeding rates, fecundity and survivorship of females (personal observation). Even though a maintenance ratio at 3:1 may carry the risk of reducing the diversity of the cage population after prolonged colonization, this risk can be minimized by periodically outcrossing the colony populations with wild type populations. Bellini et al. [28] has reported that a very minimal reduction in genetic variability is observed in an Italian *Ae. albopictus* laboratory strain by performing regular outcrosses.

Female mosquitoes require blood meals before laying eggs and they can produce eggs several times after each blood meal throughout their lifetime. Female fecundity and egg hatch rates will decrease as females age [29]. Previous studies have reported that female fecundity and egg hatch rate are highest in the first two gonotrophic cycles and then decrease after the second gonotrophic cycle [29]. In this study we only provide two blood meals to females with the aim of acquiring the maximum egg production within a short rearing period. Egg production efficiency in mosquito factories should be enhanced by reducing the number of blood meals and applied rearing cycles of a cage. The egg production after a third blood meal has been assessed and only 20% of the total egg production is achieved in the third gonotrophic cycle regardless of adult rearing densities (Additional file 3: Figure S2). Compared to a two-time blood-feeding schedule, feeding three times will increase the adult rearing cycle duration, human labor and will reduce the utilization rate of a cage and egg quality. Thus, two blood-feedings per rearing cycle are recommended for *Ae. albopictus* egg producing colonies in mosquito factories as almost 80% of possible egg production would be achieved while minimizing costs.

Artificial blood-feeding systems should be developed in any mosquito mass rearing facility. The uses of natural and artificial membranes, such as collagen sausage skins, bovine intestines (cleaned) and Parafilm, have been tested for several mosquito species [10, 18, 19, 30–32]. The blood temperature is an important parameter for feeding responses of females [33]. Blood-feeding systems with electric temperature control have also been developed for *Anopheles* mosquito mass rearing cages [18, 19]. The *Aedes* MPC with two blood-feeding slots into which collagen sausage skins filled with blood can be inserted in vertical positions, has been shown to improve the feeding rate of females compared to blood sources placed on top of the cage [18, 34]. However, the blood-feeding method for the *Aedes* MPC requires the use of a larger volume of blood compared to the traditional artificial feedings methods [18]. The average blood volume ingested by an individual female is approximately 0.7 μl, regardless of whether the blood meals contain ATP or not (Fig. 3). Hence, the volume of blood required for normal cage structures (cubes or cuboids) by using aluminum plate feeding systems is less than that required for the *Aedes* MPC, which can reduce the cost of blood considerably in a mosquito facility. The quality of blood will also affect the blood-feeding rate of females, especially for females of the *Aedes* genus, most of which do not feed on defrosted blood as it is likely that hemolysis happens after freezing. However, it has been reported that only the blood plasma plays a role in the processes of egg formation [35] and further studies should be performed on the effects of defrosted plasma on the blood-feeding of females.

To improve the blood-feeding response of females, attractants such as honey, glucose and ATP have been added to the blood meals [36, 37]. Our results have also indicated that blood supplemented with ATP can increase the blood-feeding rate of females, which is similar to previous studies [33, 37]. Higher feeding frequency can lead to higher egg production per cage, thus ATP is recommended to be added to blood meals to improve the egg production for *Ae. albopictus*. However, due to the high costs of ATP (laboratory use level), it is impossible to use it in a mosquito factory as this will increase the total costs of mass rearing. Thus finding other low cost supplements to improve the blood-feeding frequency are important for mosquito factories. Additional research is recommended to focus on the development of artificial blood meals, which has been proposed for both *Ae. albopictus* and *Ae. aegypti* [35, 38]. Compared to the use of animal blood, which should undergo testing for microbial contamination before feeding, artificial blood meals would be suitable for mosquito rearing as it might be safer in terms of quality control of both mosquitoes and insectary staff. This, however, requires more research before possible implementation [33, 38].

Compared to small adult rearing cages (e.g. 30 × 30 × 30 cm), large cages can not only hold more mosquitoes leading to higher egg production, but can also reduce requirements for rearing space, manual labor and mosquito escapes when in operation. However, cage size and design should not negatively affect female fecundity and egg hatch rate [18, 19]. In this study, we found that the cage structure had no impacts on egg hatch rate (Table 4), but affected the egg production and female fecundity (Table 4). It is interesting to note that higher egg production was achieved in Big cage B-2 compared to Big cage B-1 even though the same number of mosquitoes was used (Table 4). This difference may be due to the structure of the cage, as the lower height (30 cm) of the cage results in a higher blood-feeding rate compared to the taller height (50 cm). Thus, a good balance should be determined between the height and width of the cage aiming at finding an optimal cage structure for mosquito adult colony maintenance. The average fecundity of *Aedes* females has been found to range between 42–88 eggs per female in the first gonotrophic cycle under laboratory conditions, however, the female fecundity ranges between 30–74 eggs per female regardless of cage structure and rearing density after two blood-feedings (Tables 2–5) in this study, which is lower than reported in a previous study [39]. Even though female fecundity can be affected by many factors, such as the quality of blood, blood volume ingested by the female and female insemination status, in this study, the female fecundity is calculated as the total number of eggs divided by the number of females added to the cage, where the blood-feeding frequency and fatalities of females are neglected, resulting in a lower female fecundity in the mosquito mass rearing factory than in laboratory conditions. Carvalho et al. [10] has also reported that an average of 48 eggs per *Ae. aegypti* individual female can be acquired within two weeks (twice blood-feedings per week) in a mosquito factory. However, the description of egg production or female fecundity within one rearing cycle in each cage is much clearer for mass production. Too high rearing densities for mosquitoes in cages will decrease the egg production and female fecundity [19]. Thus, a balance should be determined between the rearing density and egg production/female fecundity for each adult rearing cage.

## Conclusions

Our study has indicated that the sex ratio, rearing density and cage structure are important parameters for mass egg production under factory conditions. High levels of egg production can be achieved using Big cage A with an introduction of 16,000 mosquitoes at 3:1 ratio of females to males within 15 days with two blood meals supplemented with ATP. Based on the cage structure and rearing schedule developed in this study, we have designed an adult rearing unit for *Ae. albopictus* with the capacity of producing 10 million eggs. Results from this study can significantly contribute to mosquito mass rearing in support of the establishment of a medium-sized mosquito facility for any genetic control strategy requiring the mass production of the target species.

## Additional files

Additional file 1: **Figure S1.** Design of experimental rearing procedures for adult *Aedes albopictus*. (TIFF 139 kb)

Additional file 2: **Table S1.** Comparison between rearing modules using small cages and Big cage A for the production of 10 million eggs of *Aedes albopictus*. (DOCX 13 kb)

Additional file 3: **Figure S2.** Percentage of egg production at different egg collection points with different adult rearing densities using Big cage A. (TIFF 159 kb)

## Abbreviations

ATP: Adenosine triphosphate
BF: Blood-feeding
DRS: Density-resting surface
EC: Egg collection
HC: Triple *Wolbachia*-infected *Aedes albopictus*
IIT: Incompatible insect technique
MPC: Mass production cage
RH: Relative humidity
SEM: Standard error of mean
SF: Sugar-feeding
SIT: Sterile insect technique
WHO: World Health Organization
