# Supplementary figures and images for: Establishment of a medium-scale mosquito facility: tests on mass production cages for Aedes albopictus (Diptera: Culicidae)
Source: Parasit Vectors. 2018 Mar 19;11:189. doi: 10.1186/s13071-018-2750-7 (PMC5859650; doi:10.1186/s13071-018-2750-7)

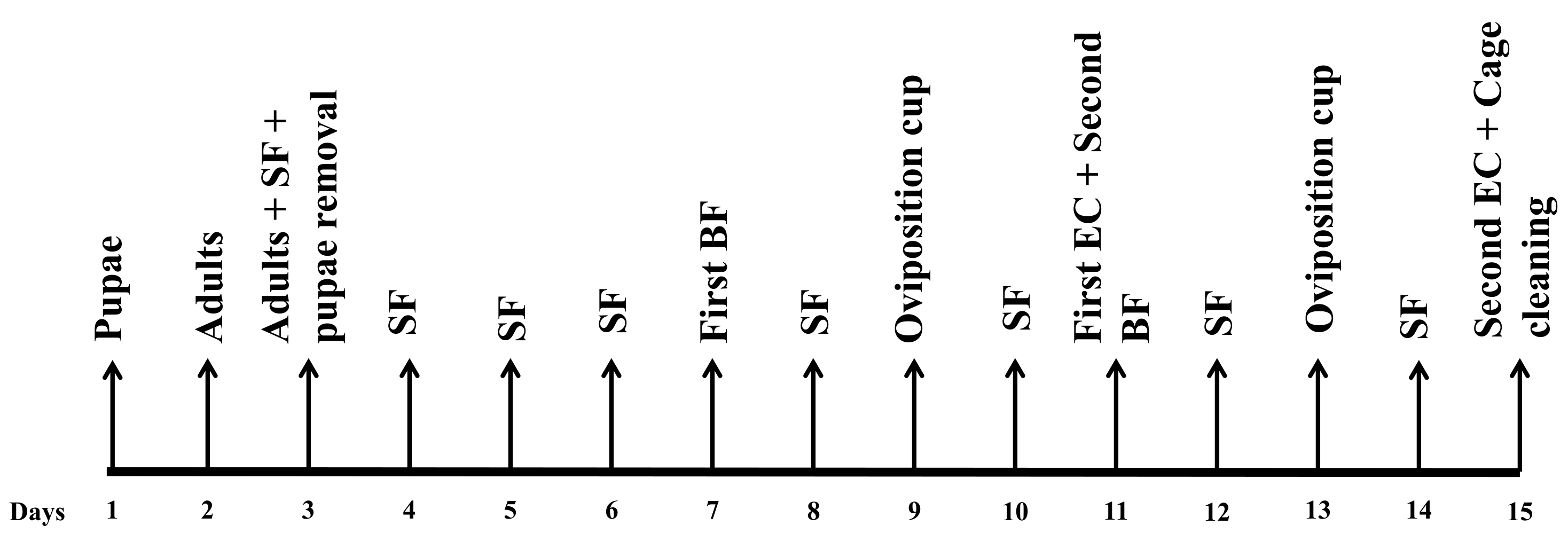

Supplement: Supplementary file 1 — Figure S1. Design of experimental rearing procedures for adult Aedes albopictus. (TIFF 139 kb) [file 13071_2018_2750_MOESM1_ESM.tif]

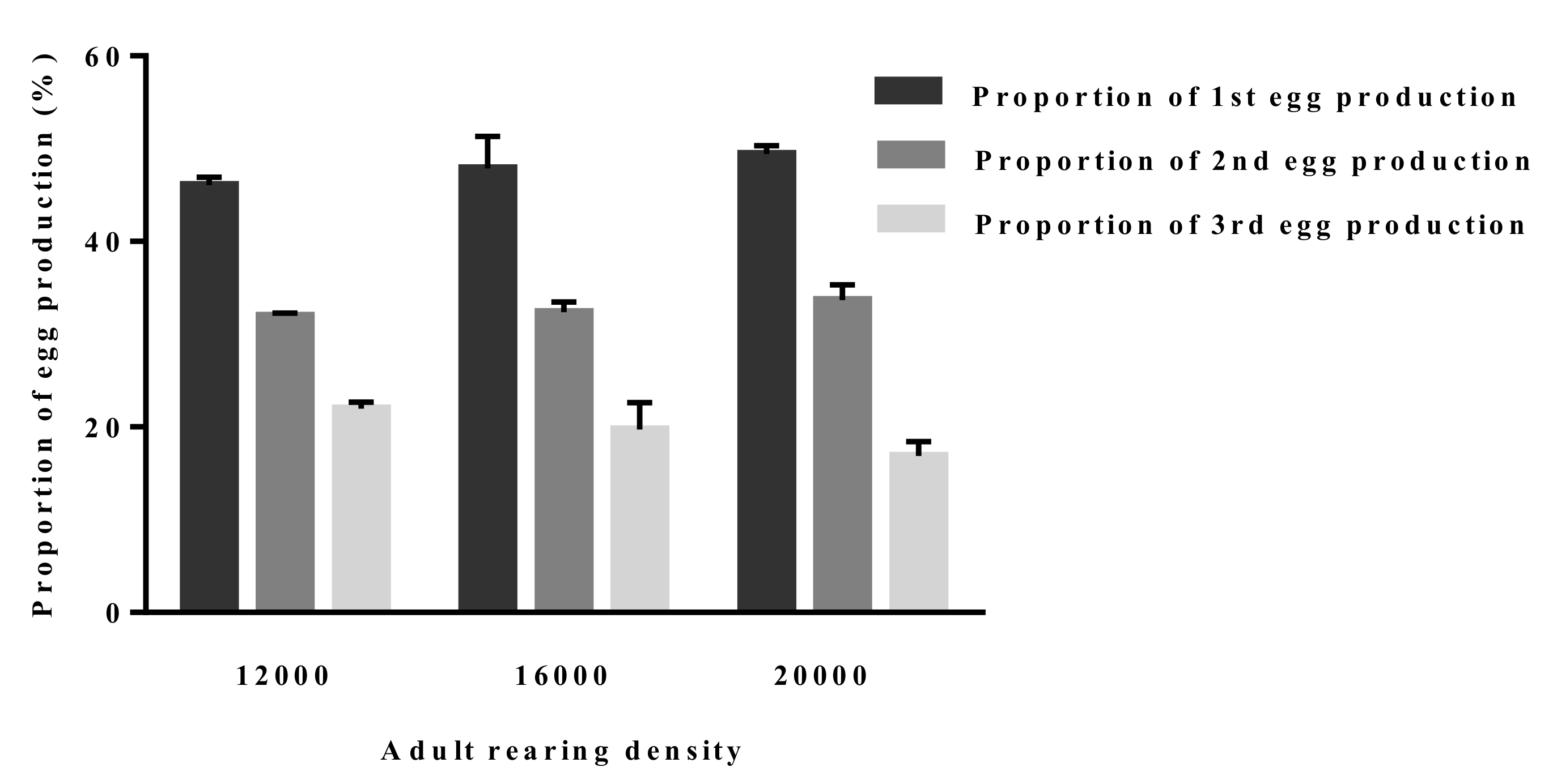

Supplement: Supplementary file 3 — Figure S2. Percentage of egg production at different egg collection points with different adult rearing densities using Big cage A. (TIFF 159 kb) [file 13071_2018_2750_MOESM3_ESM.tif]
