# Supplementary material for: Establishment of a medium-scale mosquito facility: tests on mass production cages for Aedes albopictus (Diptera: Culicidae)
Source: Parasit Vectors. 2018 Mar 19;11:189. doi: 10.1186/s13071-018-2750-7 (PMC5859650; doi:10.1186/s13071-018-2750-7)
Supplement: Supplementary file 2 — Table S1. Comparison between rearing modules using small cages and Big cage A for the production of 10 million eggs of Aedes albopictus. (DOCX 13 kb) [file 13071_2018_2750_MOESM2_ESM.docx]

**Table S1.** Comparison on adult rearing unit by Small cage and Big cage A unit for producing ten million eggs of *Aedes albopictus*

| Parameter | Small cage unit | Big cage unit |
| --- | --- | --- |
| Average egg production per cage (10^6^) | 0.19 | 0.73 |
| Cage number per unit | 12 | 15 |
| Space per unit (m^2^) | 0.3 | 1.8 |
| Egg production per unit (10^6^) | 2.28 | 10.95 |
|  |  |  |
| NO. of unit for 10 million eggs | 5 | 1 |
| NO. of cages for 10 million eggs | 60 | 15 |
| Space for 10 million eggs | 1.5 | 1.8 |
|  |  |  |
| Labor | Much | Few |
| Application | Small size facility | Medium size facility |
